# Cerebellar Functional Parcellation Using Sparse Dictionary Learning Clustering

**DOI:** 10.3389/fnins.2016.00188

**Published:** 2016-05-02

**Authors:** Changqing Wang, Judy Kipping, Chenglong Bao, Hui Ji, Anqi Qiu

**Affiliations:** ^1^Graduate School for Integrative Sciences and Engineering, National University of SingaporeSingapore, Singapore; ^2^Department of Biomedical Engineering, National University of SingaporeSingapore, Singapore; ^3^Department of Mathematics, National University of SingaporeSingapore, Singapore; ^4^Clinical Imaging Research Centre, National University of SingaporeSingapore, Singapore; ^5^Singapore Institute for Clinical Sciences, Agency for Science, Technology, and ResearchSingapore, Singapore

**Keywords:** resting-state functional magnetic resonance imaging, sparse coding, dictionary learning, cerebellum, brain parcellation

## Abstract

The human cerebellum has recently been discovered to contribute to cognition and emotion beyond the planning and execution of movement, suggesting its functional heterogeneity. We aimed to identify the functional parcellation of the cerebellum using information from resting-state functional magnetic resonance imaging (rs-fMRI). For this, we introduced a new data-driven decomposition-based functional parcellation algorithm, called Sparse Dictionary Learning Clustering (SDLC). SDLC integrates dictionary learning, sparse representation of rs-fMRI, and k-means clustering into one optimization problem. The dictionary is comprised of an over-complete set of time course signals, with which a sparse representation of rs-fMRI signals can be constructed. Cerebellar functional regions were then identified using k-means clustering based on the sparse representation of rs-fMRI signals. We solved SDLC using a multi-block hybrid proximal alternating method that guarantees strong convergence. We evaluated the reliability of SDLC and benchmarked its classification accuracy against other clustering techniques using simulated data. We then demonstrated that SDLC can identify biologically reasonable functional regions of the cerebellum as estimated by their cerebello-cortical functional connectivity. We further provided new insights into the cerebello-cortical functional organization in children.

## 1. Introduction

The human cerebellum is a structure located underneath the cerebral hemispheres. It consists of more than 50% of neurons in the brain even though it takes up only approximately 10% of the total brain volume. For a long period of time, the cerebellum was thought to solely contribute to the planning and execution of movement, until quite recently, where new discovery revealed that the majority of the cerebellum is associated with cerebral networks involved in emotion, language, attention, and mental imagery etc. (Stoodley and Schmahmann, 2009; Strick et al., 2009; Schmahmann, 2010; Buckner, 2013). This suggests that the cerebellum is functionally heterogeneous. Nevertheless, a comprehensive picture on functional zones of the human cerebellum is largely unavailable.

In the last decade, resting-state functional magnetic resonance imaging (rs-fMRI) has emerged as a useful imaging technique for mapping intrinsic functional organization of the brain. The cerebral cortex has been divided into different functional regions that were inferred by measuring the pattern of spontaneous low-frequency fluctuations of rs-fMRI signals (Fransson, 2005; Greicius et al., 2009; Yeo et al., 2011). This demonstrates that rs-fMRI is a surprisingly powerful approach for comprehensively understanding the functional regions of the brain. Recently, rs-fMRI has also been used to study the functional organization of the cerebellum. Most existing studies identified the cerebellar functional representation based on its functional connectivity with predefined cortical regions (Krienen and Buckner, 2009; O'Reilly et al., 2010; Buckner et al., 2011). However, the cerebellum may have striking functional networks different from the cerebral cortex, which may not be discovered using the cortical-region-driven approach. Hence, studying the pattern of the rs-fMRI signal fluctuation within the cerebellum might allow for a more accurate representation of cerebellar functional organization (Bernard et al., 2012; Dobromyslin et al., 2012).

Data-driven clustering and decomposition approaches have been widely used to parcellate the functional zones of individual structures using rs-fMRI (Li et al., 2009). Cluster analysis uses the rs-fMRI signals at every time point as features for clustering. Some examples of cluster analysis methods used to study functional connectivity are fuzzy clustering (Golay et al., 1998), hierarchical clustering (Cordes et al., 2002) and normalized cut/spectral clustering (Shen et al., 2010, 2013). Additional spatial constraints can be imposed on cluster analysis methods to enhance spatial contiguity of the clusters (Craddock et al., 2012; Blumensath et al., 2013). Clustering can also be done using connectivity patterns as features (Yeo et al., 2011; Eickhoff et al., 2015).

Decomposition-based methods on the other hand first perform blind source separation (BSS) on the fMRI signals, to obtain a set of source signals and a matrix of coefficients that describe how the original fMRI signal can be reconstructed from the source signals. Subsequently functional connectivity can be determined from the matrix of coefficients by thresholding the matrix, or by performing clustering on the matrix using the coefficients as features. BSS is a reasonable approach because the fMRI time series recorded at each voxel contains a mixture of signals, including neuronal and physiological oscillations, as well as motion or machine artifacts (McKeown et al., 1997). A commonly used BSS technique is independent component analysis (ICA). The source signals obtained with ICA are either spatially or temporally independent (McKeown et al., 1997; Beckmann et al., 2005; Smith et al., 2012). Another possible BSS technique is non-negative matrix factorization (NNMF) (Sotiras et al., 2015).

An emerging BSS technique that shows great promise is dictionary learning (Mairal et al., 2009; Varoquaux et al., 2011). The set of source signals (dictionary elements) obtained from dictionary learning may be over-complete, and hence the matrix of coefficients could be sparse. Lee et al. (2011) suggest that the sparsity constraint of dictionary learning is more suitable for the BSS of fMRI than the independence constraint of ICA. Spatial independence biases ICA toward finding relatively sparse as well as discrete connected components, since components that span large areas, or that overlap with other components, have a tendency to be split into multiple separate components (McKeown et al., 1997). In addition, sparse coding has been observed in the brain (Olshausen and Field, 1996; Quiroga et al., 2008). Lee et al. (2011) and Li et al. (2012) both applied dictionary learning on fMRI data for the sparse representation of the brain. Li et al. did so with the purpose of denoising the fMRI signals. However, both did not seek to parcellate the brain. Abraham et al. (2013) performed parcellation of the brain by thresholding the spatial maps of the dictionary elements, so that on average, each voxel is non-zero in only one of the maps. This means that the parcellation obtained would be very fine, as the number of functional regions is proportional to the number of dictionary elements. Furthermore, the possibility that a functional region could consist of more than one dictionary element is excluded.

This paper proposes a new method for performing functional parcellation in the cerebellum, called Sparse Dictionary Learning Clustering (SDLC). As its name suggests, the proposed method is based on dictionary learning, and hence it has all the advantages over other decomposition-based techniques as mentioned earlier. The proposed method incorporates dictionary learning, sparse representation, and clustering of fMRI data into discrete regions. The proposed method is explained in detail in Section 2. Section 3 describes and shows the results of the experiments using simulated data that were conducted on the proposed method, to test and benchmark it against existing techniques. SDLC was also applied to real rs-fMRI data of the cerebellum where we expected a segregation into multiple motor and non-motor regions. We tested this by measuring the functional connectivity between the cerebellar clusters with the neocortex. We expect to find connectivity to well-known sensorimotor regions, but at the same time we also aim to identify the less understood cerebellar involvement in non-motor systems, specifically in young children.

## 2. Materials and methods

### 2.1. MRI acquisition and preprocessing

Fifty-eight subjects were selected from an existing study on cognition and brain development in children (mean age: 77.4 months, standard deviation 3.8 months) (Qiu et al., 2012; Zhong et al., 2014). Written consent was obtained from participants parents under the approval of the Institutional Review Board of the National University of Singapore (NUS).

The MRI data of the subjects were acquired on a 3T Siemens Magnetom Trio Tim scanner using a 32-channel head coil at the Clinical Imaging Research Centre at the National University of Singapore (NUS). The image protocols were: (i) high-resolution isotropic T1-weighted Magnetization Prepared Rapid Gradient Recalled Echo (MPRAGE; 190 slices, 1 mm thickness, in-plane resolution 1 mm, no inter-slice gap, sagittal acquisition, field of view 190 × 190 mm, matrix = 190 × 190, repetition time = 2000 ms, echo time = 2.08 ms, inversion time = 850 ms, flip angle = 90°); (ii) isotropic axial rs-fMRI imaging protocol (single-shot echo-planar imaging; 42 slices with 3 mm slice thickness for the whole brain coverage, no inter-slice gaps, matrix = 64 × 64, field of view = 190 × 190 mm, repetition time = 2400 ms, echo time = 27 ms, flip angle = 90°, scanning time = 6) min. The children were asked to close their eyes during the rs-fMRI scan.

The rs-fMRI data were first processed with slice timing, motion correction, skull stripping, band-pass filtering (0.01–0.08 Hz) and grand mean scaling of the data (to whole brain modal value of 100). All the rs-fMRI datasets used in this study had framewise displacement less than 0.5 mm (Power et al., 2012). Effects of nuisance variables, including detrending of rs-fMRI, six parameters obtained by motion correction (3 rotation parameters and 3 translation parameters), ventricular and white matter signals, were reduced by means of regression. Only the first order of these regressors were considered in the regression analysis. Subsequently, the individual rs-fMRI data were registered to the atlas space based on the transformation from the individual T1-weighted image to the atlas via large deformation diffeomorphic metric mapping (LDDMM) (Du et al., 2011). The rs-fMRI data was visually inspected to ensure that they have full cerebellar coverage. Finally, we extracted rs-fMRI signals from the gray matter by using a binary gray matter mask obtained from the tissue segmentation in FreeSurfer (Fischl et al., 2002). The cerebellar rs-fMRI data were spatially smoothed with a Gaussian kernel with a full width at half maximum (FWHM) of 6 mm to increase signal to noise ratio (SNR).

### 2.2. Sparse dictionary learning clustering (SDLC) for cerebellar parcellation

We denote the rs-fMRI time series in the cerebellar gray matter region as *Y* = [*Y*_1_, *Y*_2_, ⋯, *Y*_*i*_, ⋯, *Y*_*n*_υ__], where $Y_{i}={\left[y_{ji}\right]}_{j=1}^{n_{t}}$ is the rs-fMRI time series of the *i*^*th*^ voxel with *n*_*t*_ time points. We assume that *Y*_*i*_ can be characterized by a few elements in a dictionary *D*, comprised of time course exemplars. We represent $D\in {\mathbb{R}}^{n_{t}\times n_{d}}$ as a matrix, where column *D*_*i*_ is an element of the dictionary with *n*_*t*_ time points, for a total of *n*_*d*_ elements. We expect that voxels in the cerebellum with similar waveforms of the time series can be described by a similar set of the dictionary elements and hence result in being classified into the same functional unit of the cerebellum. Therefore, we propose a sparse dictionary learning clustering (SDLC) algorithm to parcellate the cerebellum into functional units based on the rs-fMRI time series data. As its name suggests, the SDLC incorporates (1) dictionary learning on the collection of rs-fMRI time series from all voxels in the cerebellum to seek an dictionary of the time series exemplars and sparse representation of real rs-fMRI data; (2) a classifier that groups voxels represented with similar dictionary elements as one functional unit. To achieve this, we formulate an optimization problem for the SDLC as

(1) $$\begin{array}{lll} \underset{D,L,S}{\mathrm{argmin}}\; & \;\|Y-DS{\|}_{F}^{2}+\alpha \|S{\|}_{1}+\beta \|S\left(I-L\right){\|}_{F}^{2}\;,\; & \; \end{array}$$

$$\begin{array}{lll} subject to\; & \;\forall i\;,\;\|D_{i}{\|}_{F}=1\;;\;L\neq I\;;\;L=\overset{n_{l}}{\underset{i=1}{\sum }}\frac{1_{i}\times 1_{i}^{T}}{\sum 1_{i}}\;,\; & \; \end{array}$$

where *L* contains the cluster information for every voxel and has a dimension of (*n*_υ_ × *n*_υ_), 1_*i*_ is the indicator function for cluster *i* for a total of *n*_*l*_ clusters and has a dimension of (*n*_υ_ × 1), and *T* is the matrix transpose operator. ‖ · ‖_*F*_ and ‖ · ‖_1_ are the matrix Frobenius and ℓ_1_ norms respectively. $S\in {\mathbb{R}}^{n_{d}\times n_{\upsilon }}$ is the dictionary coefficients, where each column of *S* is the coefficients of all *n*_*d*_ dictionary elements for a particular voxel, for a total of *n*_υ_ voxels. *I* is the identity matrix. The columns of *S*−*SL* are the distances between each voxel and its nearest cluster center. Hence, minimizing the Frobenius norm of *S*−*SL* is equivalent to performing k-means clustering. α and β are user-defined weights of the second and third terms in Equation (1) respectively. We discuss how to set these parameters below. Here, the second and third terms in Equation (1) seem redundant but both are necessary and one facilitates the other. Our aim is to minimize Equation (1) with respect to *L*, *S*, and *D*.

To minimize the cost function in Equation (1), we adapt a multi-block hybrid proximal alternating method that is empirically fast and guarantees strong convergence (see theoretical proof in Bao et al., 2015). In this method, solving Equation (1) is equivalent to iteratively solving the following three optimization problems.

(2) $$\begin{array}{r} \underset{L}{\mathrm{argmin}}\;\beta \|S\left(I-L\right){\|}_{F}^{2}+\gamma_{1}\|L-L^{k}{\|}_{F}^{2}\;, \end{array}$$

$$\text{subject to }L\neq I\;;\;L=\sum_{i=1}^{n_{l}}\frac{1_{i}\times 1_{i}^{T}}{\sum 1_{i}}\;.$$

(3) $$\underset{S}{\text{argmin}}\;\|Y-DS{\|}_{F}^{2}+\beta \|S(I-L){\|}_{F}^{2}+\alpha \|S{\|}_{1}+\gamma_{2}\|S-S^{k}{\|}_{F}^{2}\;.$$

(4) $$\begin{array}{r} \underset{D}{\mathrm{argmin}}\;\|Y-DS{\|}_{F}^{2}+\gamma_{3}\|D-D^{k}{\|}^{2}\;, \end{array}$$

$$\begin{array}{r} \text{subject to }\forall i\;,\;\|D_{i}{\|}_{F}=1\;. \end{array}$$

*S*^*k*^, *L*^*k*^ and *D*^*k*^ are the solutions of *S*, *L* and *D* respectively at the *k*th iteration.

Firstly, we solve *L* via k-means clustering by minimizing the cost function in Equation (2). The columns of *S* are used as the features of the voxels for clustering. The k-means clustering is initialized using the cluster information obtained in the previous iteration.

Secondly, optimizing *S* is equivalent to minimizing the cost function in Equation (3) when *L* and *D* are known. We can restate Equation (3) as the sum of G(*S*) and H(*S*), where

$$G\left(S\right)=\|Y-DS{\|}_{F}^{2}+\beta \|S\left(I-L\right){\|}_{F}^{2}+\gamma_{2}\|S-S^{k}{\|}_{F}^{2}\;,$$

and

$$H\left(S\right)=\alpha \|S{\|}_{1}\;.$$

G(*S*) is a differentiable convex function with Lipschitz continuous gradient, whereas H(*S*) is a non-smooth but convex function. We adopt a fast computation algorithm, accelerated proximal gradient (APG), to solve the above ℓ_1_-norm optimization problem (Toh and Yun, 2010; Bao et al., 2012). As shown in Algorithm 2.1, APG requires several inputs, including the convex function of H(*S*), an appropriate Lipschitz constant of *K*, two user-defined parameters of ρ and β, as well as the first derivative of G(*S*), $G^{\prime }\left(S\right)=-2D^{T}\left(Y-DS\right)+2\beta {\left(I-L\right)}^{T}\left(I-L\right)S+\gamma_{2}\left(S-S^{k}\right)$. The appropriate Lipschitz constant *K* needs to be selected to prevent the APG algorithm from converging slowly or even diverging. Let *K*_*i*_ be the Lipschitz constant for a particular voxel *i*. *K*_*i*_ is the largest eigenvalue of the Hessian of G(*S*_*i*_), where *S*_*i*_ is the *i*th column of *S*. *K*_*i*_ is hence the sum of the largest eigenvalues of 2*D*^*T*^*D* and $2\beta {\left(I-L\right)}_{i}{\left(I-L\right)}_{i}^{T}$, where (*I*−*L*)_*i*_ is the *i*th row of (*I*−*L*). We choose the most stringent Lipschitz constant, that is, let *K* be the smallest *K*_*i*_. In addition to *K*, β is set to be proportional to the certainty of *L*, which is measured by the sum of point-to-centroid distances during k-means clustering. A larger β would cause the estimated dictionary coefficients of voxels in the same cluster to be more similar to each other, which would in turn help SDLC to converge faster. But this is only desirable if there is certainty that *L* is accurate, hence the way β is set. We define density function F(*S*) as the ratio of the number of non-zero terms in *S* to the total number of terms in *S*. *S* is iteratively optimized via the APG algorithm with different values of α, until F(*S*) is equal to a specified value ρ. During each iteration, $\underset{c}{\mathrm{argmin}}\frac{K}{2}\|c-b_{i+1}+\frac{G^{\prime }\left(b_{i+1}\right)}{K}{\|}_{F}^{2}+H\left(c\right)$ in Algorithm 2.1 can be easily solved when replaced with $T_{\alpha ∕L}\left(b_{i+1}+\frac{G^{\prime }\left(b_{i+1}\right)}{K}\right)$, where T_·_(·) is the soft thresholding operator, i.e., $T_{\alpha ∕L}\left(b_{i+1}+\frac{G^{\prime }\left(b_{i+1}\right)}{K}\right)=\mathrm{sign}\left(b_{i+1}+\frac{G^{\prime }\left(b_{i+1}\right)}{K}\right){\left(|b_{i+1}+\frac{G^{\prime }\left(b_{i+1}\right)}{K}|-\frac{\alpha }{L}\right)}_{+}$.

**Algorithm 1:**
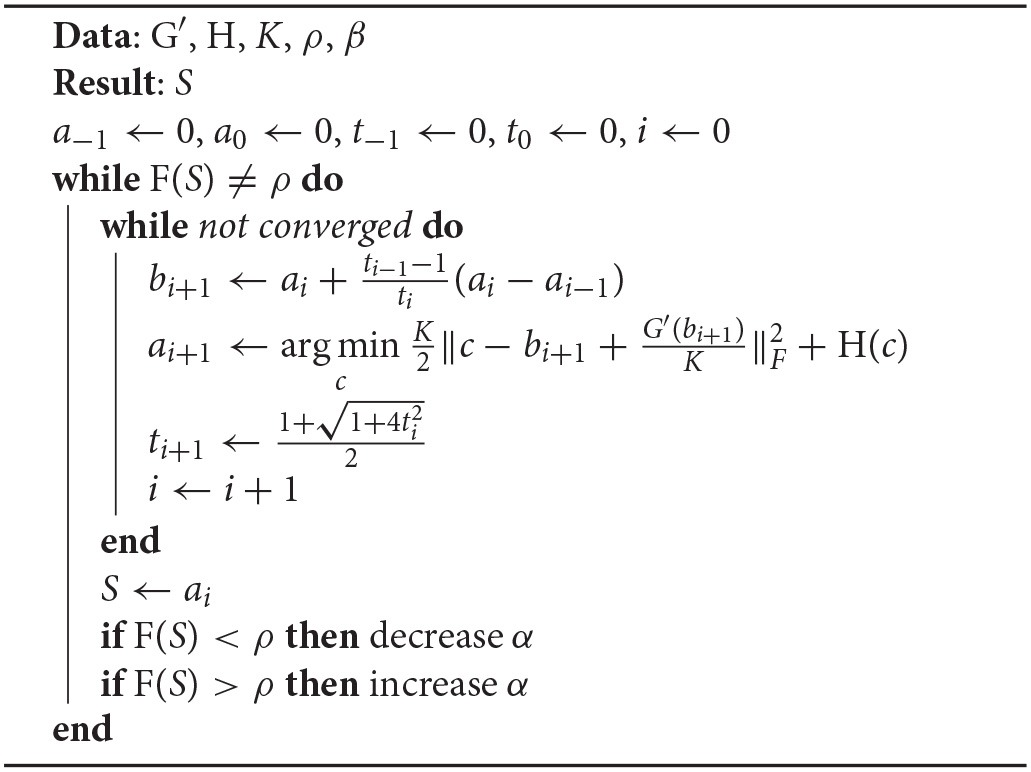
Optimizing S (APG)

Lastly, given *S*, we seek *D* via the minimization problem in Equation (4). We update $D_{i}=\frac{{\left[(YS^{T}-\gamma_{3}D^{k}){\left(SS^{T}-\gamma_{3}I\right)}^{-1}\right]}_{i}}{\|{\left[(YS^{T}-\gamma_{3}D^{k}){\left(SS^{T}-\gamma_{3}I\right)}^{-1}\right]}_{i}{\|}_{F}}$, where [*A*]_*i*_ is the *ith* column of matrix *A*. Hence, the SDLC algorithm is comprised of solving the optimization problems in Equations (2, 3, 4). The entire process is repeated until *S*, *L* and *D* converge.

### 2.3. Initialization of the SDLC algorithm

To start the optimization algorithm of SDLC outlined in the above section, it is crucial to initialize *S*, *L* and *D*. In our study, *D* is initialized by randomly selecting voxels from the cerebellum of multiple subjects, and using the time series of these voxels as dictionary elements, after they have been centered and normalized. *S* is initialized as *D*^+^*Y* where *D*^+^ = *VΣ*^+^*U*^*T*^ is the pseudo inverse of *D* with *D* = *UΣV*^*T*^ obtained using SVD. Finally, *L* is initialized by performing k-means clustering on *S*, after the columns of *S* have been normalized. The columns of *S* are used as the features of the voxels for clustering. One way to prevent the k-means clustering from falling into a local minimum, is to properly initialize it with a good guess of the clusters, by consulting existing parcellations. Alternatively, in our study, k-means clustering is repeated for multiple times, and the instance with the lowest sum of point-to-centroid distances is used to initialize *L*.

### 2.4. Estimation of the number of clusters

We employed stability analysis (Lange et al., 2004; Buckner et al., 2011) to determine how many clusters there should be for the k-means clustering step of SDLC. We conducted stability analysis for the left and right cerebellum separately, for each of the 58 subjects, hence for a total of 116 times. The steps of stability analysis are as follows. The voxels being clustered are divided into two groups of equal size. Both groups of voxels are put through SDLC separately (with a pre-defined number of clusters) to obtain two sets of parcellations, one for each group of voxels. Then, the first group of voxels is used as a training set—together with the label information obtained through SDLC earlier—to reclassify the second group of voxels. In this way, the second group of voxels would have two different parcellation results: one from SDLC directly, and the other predicted from the first group of voxels. Stability refers to how similar the two parcellation results of the second group of voxels are. In order to measure stability, the clusters in the first parcellation have to be matched to the clusters in the second parcellation. To do this, a weight matrix of Hamming distances between the clusters in the two parcellations was constructed, and the Hungarian algorithm (Kuhn, 1955)—which performs minimum weighted bipartite matching—was then used to find the best cluster matching that has the lowest total Hamming distance. This total Hamming distance is also known as the instability of the clustering. If the parcellation for a particular number of clusters is highly unstable, the parcellation is not robust and highly dependent on the set of voxels chosen. An overly high number of clusters leads to an arbitrary splitting of the voxels, while an overly low number leads to merging of clusters that should otherwise be kept separate (Lange et al., 2004). In our study, the number of clusters tested was ranged from 2 to 20. An instability plot can be generated, plotting the total Hamming distance along the y-axis against the number of clusters along the x-axis. For each cerebellar hemisphere of each subject, we repeated the above steps 30 times where each time the voxels being clustered are split into the two groups randomly. The 30 instability plots obtained are averaged to create one reliable instability plot for each cerebellar hemisphere of each subject.

**Figure 1:**
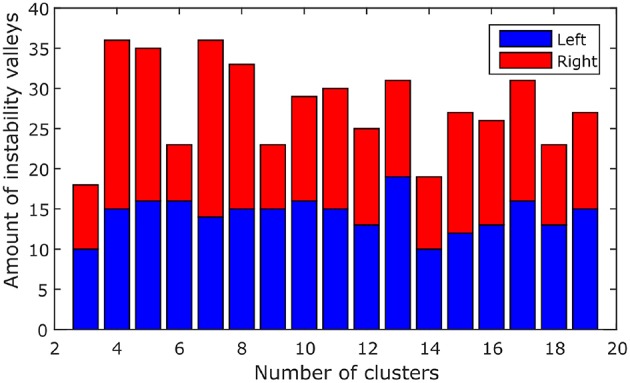
**The amount of valleys that are found at each number of clusters among 58 subjects**. Blue, left hemisphere; red, right hemisphere.

Instability valleys can be observed from the instability plot. These are points along the plot that have lower instability values than neighboring points. The positions of the valleys would indicate favorable numbers of clusters that exhibit relatively stable clustering results. To compile all 116 instability plots (from the left and right hemispheres of 58 subjects) into a single bar graph, the positions of the valleys in each of the instability plots were noted down, and the number of instability plots with valleys at each number of clusters were counted, which is shown in Figure 1. As shown in Figure 1, the numbers of clusters where most of the instability plots among 58 subjects have instability valleys at are four and seven. For this study, the number of clusters *n*_*l*_ to parcel the cerebellum into was chosen to be seven, to get a finer parcellation of the cerebellum. In addition, seven cerebellar parcels were also suggested by the one derived from the cortical parcellation (Buckner et al., 2011). Furthermore, the seven cerebellar parcels obtained in our study are also biologically meaningful as discussed below.

## 3. Results

In this section, we first evaluate the SDLC algorithm using simulated data and subsequently demonstrate its use to parcellate the cerebellum. Simulation is the only reliable way to validate the proposed method since there is no ground truth for functional parcellation. This is especially so for the parcellation of the cerebellum, as corroboration from DTI, cytoarchitecture, or even task-based fMRI, are unavailable. We concede that this is a limitation of this study. Finally, we employ the cerebellar parcellation to explore cerebello-cortical connectivity networks.

### 3.1. Simulation to demonstrate SDLC reliability

SDLC was applied on simulated data to demonstrate its reliability against noise, and to benchmark it against other existing techniques. We created a simulated dataset by first using the software SimTB (Erhardt et al., 2012) to randomly generate seven rs-fMRI time courses. Then, four arbitrary square regions were defined, and the time series for each region is calculated by combining two of the seven aforementioned time courses in particular proportions, as listed in Table 1. Regions 1 and 2 consist of two time courses each that are unique to these regions. Regions 3 and 4 also consist of two time courses each, but they both share time course six in the proportion of 0.75. This makes regions 3 and 4 harder to be distinguished from one another, especially after noise is added. The regions were designed this way so as to test the sensitivity of SDLC at distinguishing highly similar regions.

**Table 1:** **Proportions of the seven time courses in the four regions**.

	**TC1**	**TC2**	**TC3**	**TC4**	**TC5**	**TC6**	**TC7**
Region 1	0.5	0.5	0	0	0	0	0
Region 2	0	0	0.5	0.5	0	0	0
Region 3	0	0	0	0	0.25	0.75	0
Region 4	0	0	0	0	0	0.75	0.25

Each of the four square regions has 100 simulated pixels (10 × 10 pixels). The four regions are arranged into a single 20 × 20 square image, with regions 1 to 4 at the top left, top right, bottom left and bottom right respectively. The regions were also assigned colors (red, green, blue and yellow respectively) for easier visualization of the parcellation results. The time series of each pixel was calculated by combining the seven randomly generated rs-fMRI time courses according to the proportions stated in Table 1, depending on which region the pixel belongs to, but with the proportions perturbed by Gaussian noise. This is a reasonable way to generate simulated fMRI time series, because fMRI time series are the sum of signals from multiple sources (Biswal and Ulmer, 1999), and voxels within the same functional cluster could comprise the same constituent signals but in differing amounts. Signal to noise ratio in this study was defined as $\mathrm{SNR}=\bar{S}∕\sigma$, where $\bar{S}=0.143$ is the mean of all the values in Table 1, and σ is the standard deviation of the Gaussian noise used to perturb the proportions when generating the time series of individual pixels.

We employed SDLC, as well as other basic methods used in rs-fMRI studies, on the simulated data. In the cluster analysis category, methods included were hierarchical clustering with ward linkage (HCWL) (Dimitriadou et al., 2004; Thirion et al., 2014), k-means clustering (Dimitriadou et al., 2004; Thirion et al., 2014), and multi-class spectral clustering (MSC) (Thirion et al., 2014). The time points in the fMRI time series were used as features for the cluster analysis methods, therefore, no spatial constraints were imposed. In the decomposition-based category, methods included were spatial ICA (sICA) and temporal ICA (tICA) (McKeown et al., 1997; Beckmann et al., 2005; Smith et al., 2012). The ICA parcellations were obtained by first using FastICA to decompose the time series into independent components and their respective spatial maps, and subsequently k-means clustering was performed on these spatial maps.

**Figure 2:**
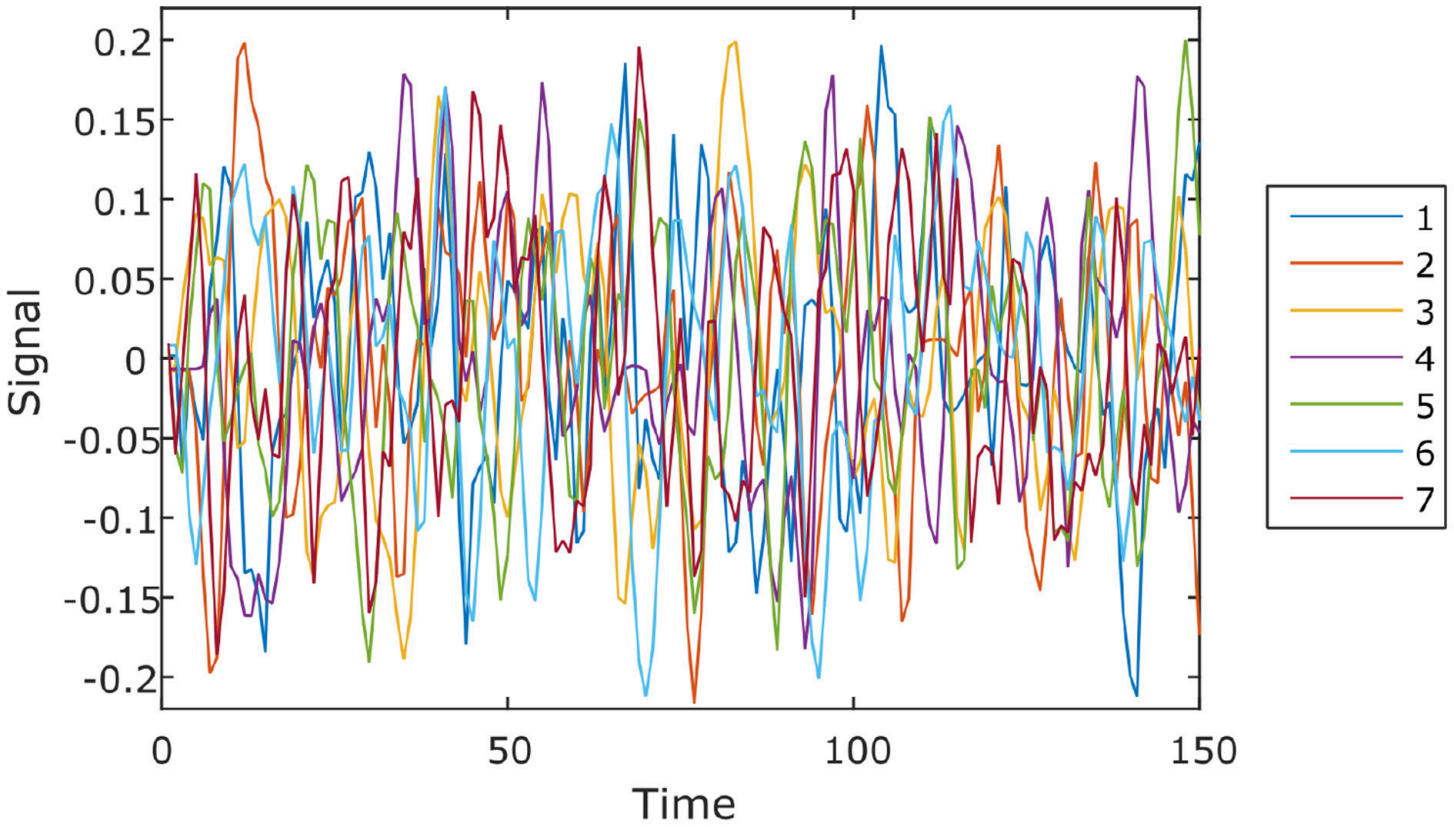
**Seven rs-fMRI time courses generated by SimTB for the first simulation**.

**Figure 3:**
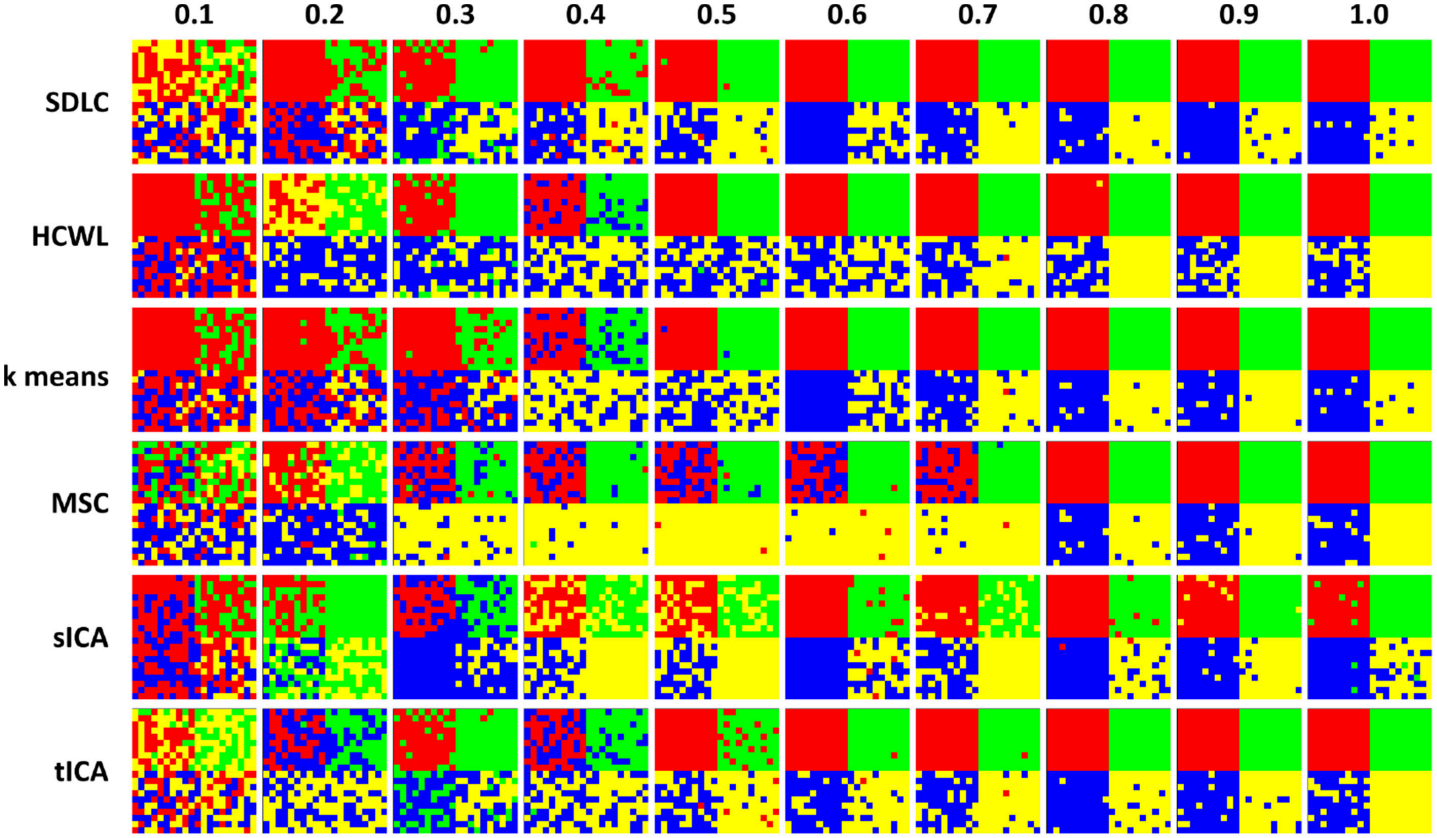
**Clustering results for the first simulation**. Each row is the clustering results for each of the six techniques tested, namely: SDLC, hierarchical clustering with ward linkage (HCWL), k-means clustering, multi-class spectral clustering (MSC), spatial ICA (sICA) and temporal ICA (tICA). Signal to noise ratio of each column is labeled at the top of the figure. Columns from left to right have increasing signal to noise ratios.

**Figure 4:**
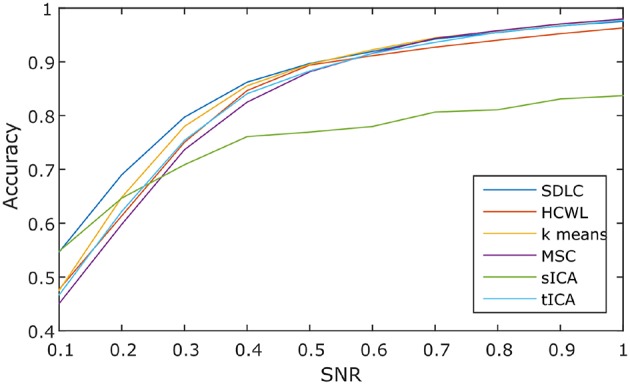
**Clustering accuracy against signal to noise ratio of the six techniques tested over 100 simulations, namely: SDLC, hierarchical clustering with ward linkage (HCWL), k-means clustering, multi-class spectral clustering (MSC), spatial ICA (sICA) and temporal ICA (tICA)**.

We ran the simulation 100 times, each time with a different set of seven rs-fMRI time courses generated by SimTB, and noise added. The seven time courses of the first simulation are plotted in Figure 2. Figure 3 shows the clustering results of the first simulation for each method at each signal-to-noise level. Figure 4 plots the overall clustering accuracy of all six methods over the 100 simulations. As can be seen from Figure 4, other than sICA, the other five techniques have fairly similar accuracy rates. However, we can see that SDLC outperforms the other techniques as SNR drops below 0.5. This simulation hence shows the advantage of using SDLC for functional parcellation in the case where functional clusters do indeed comprise the same constituent signals but in differing amounts.

### 3.2. Cerebellar parcellation

**Figure 5:**
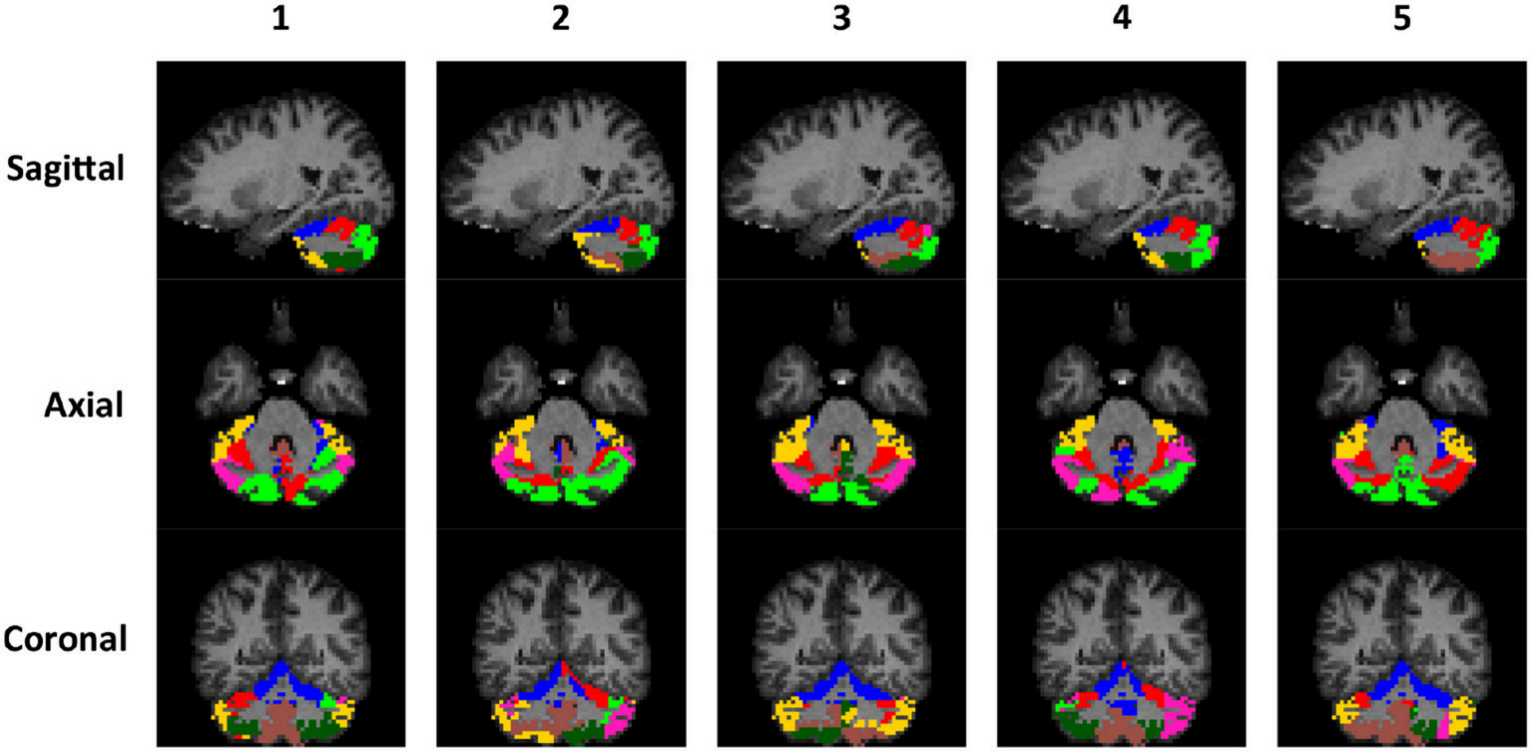
**Individual examples of the cerebellar parcellation into seven clusters**. Each column shows the cerebellar parcellation of one subject in the sagittal, axial, and coronal view.

The rs-fMRI data of the left and right cerebellum of each subject were put through SDLC separately. The order that the voxels appear in *Y* does not matter because SDLC does not impose spatial constraints when performing functional parcellation. The time series of the voxels in the cerebellum were concatenated into matrix *Y*. There were *n*_*t*_ = 150 time points in each time series, and there were *n*_υ_ = 2124 voxels in the left cerebellum and *n*_υ_ = 2237 voxels in the right cerebellum. The dictionary was initialized with *n*_*d*_ = 300 elements, and the sparsity of *S* was constrained at 5%. Figure 5 shows the individual parcellation results of five subjects.

**Figure 6:**
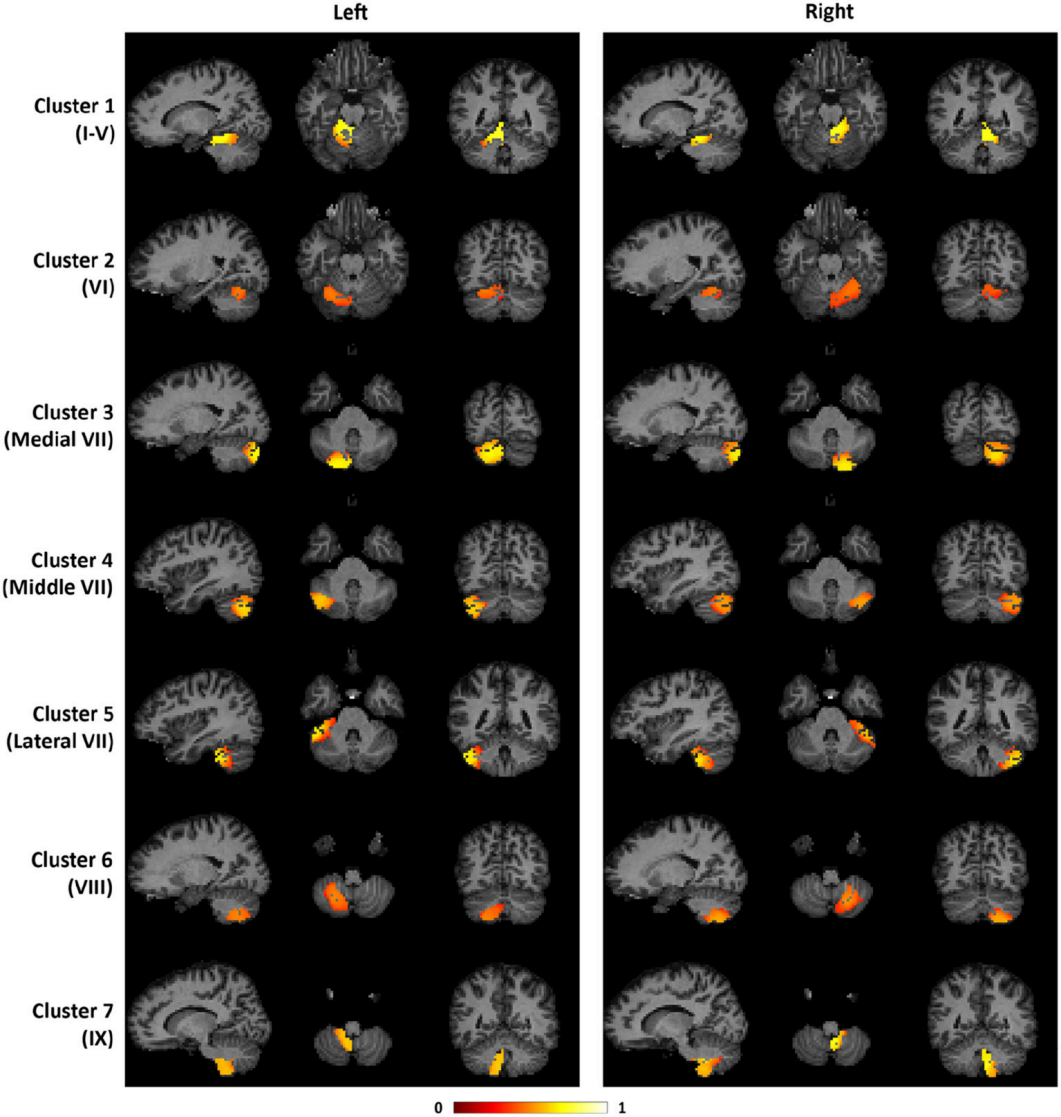
**The probability maps for the seven cerebellar clusters**. The left half of the figure displays the seven clusters in the left cerebellum, while the right half displays the seven clusters in the right cerebellum. From top to bottom, the seven clusters are located in the following anatomical lobules: I-V; VI; Medial VII; Middle VII; Lateral VII; VIII; IX.

To illustrate the consistency of the cerebellar parcellation across all 58 subjects, we aggregated the cerebellar parcellation of individuals into a group parcellation in the atlas space. We represent the aggregated results based on probability maps, one map for each cerebellar cluster. The map of each cluster shows the probability of every cerebellar voxel belonging to that particular cluster, as opposed to the other clusters. The cerebellar voxels were then assigned to the cluster that they most likely belong to, while still retaining the probability information. The group parcellation is shown in Figure 6. Although the hemispheres were separately clustered using SDLC, a promising indication for the validity of the group parcellation is the high symmetric pattern between each of the seven left and right hemispheric clusters. Nevertheless, we notice that the size of some clusters in the left hemisphere may not be equal to that in the right hemisphere, which is consistent with that seen in adults (Buckner et al., 2011).

### 3.3. Cerebello-cortical connectivity maps

In this section, we aimed to identify cerebello-cortical connectivity patterns, which can further reinforce the validity of the cerebellar parcellation obtained using SDLC. For this, the first level analysis of finding cerebello-cortical connectivity was performed at the subject level. Using the cerebellar parcellation obtained earlier, a representative time series for each cluster was calculated by taking the mean of the time series of all the voxels in the cluster. Taking these 14 time series data (seven per cerebellar hemisphere) as independent variables, General Linear Model (GLM) fitting was done for every voxel in the cerebral cortex separately, using the time series of each cortical voxel as the dependent variable. The β_*GLM*_ obtained for every cortical voxel from the GLM fitting was spatially smoothed in the volume using a Gaussian kernel with FWHM of 6mm. This was repeated for all 58 subjects. The second level analysis of finding cerebello-cortical connectivity was done at the group level. Student's *t*-test was performed on the β_*GLM*_ obtained earlier for every cortical voxel and every cerebellar cluster, across the 58 subjects with the null hypothesis of β_*GLM*_ = 0 and the alternative of β_*GLM*_ > 0. Voxelwise *p*-values < 0.01 with cluster size >30 (corresponding to cluster corrected *p*-value < 0.05) was considered to be significant. Cluster size threshold was obtained using AFNI's 3dClustSim (Version 16.0.18).

**Figure 7:**
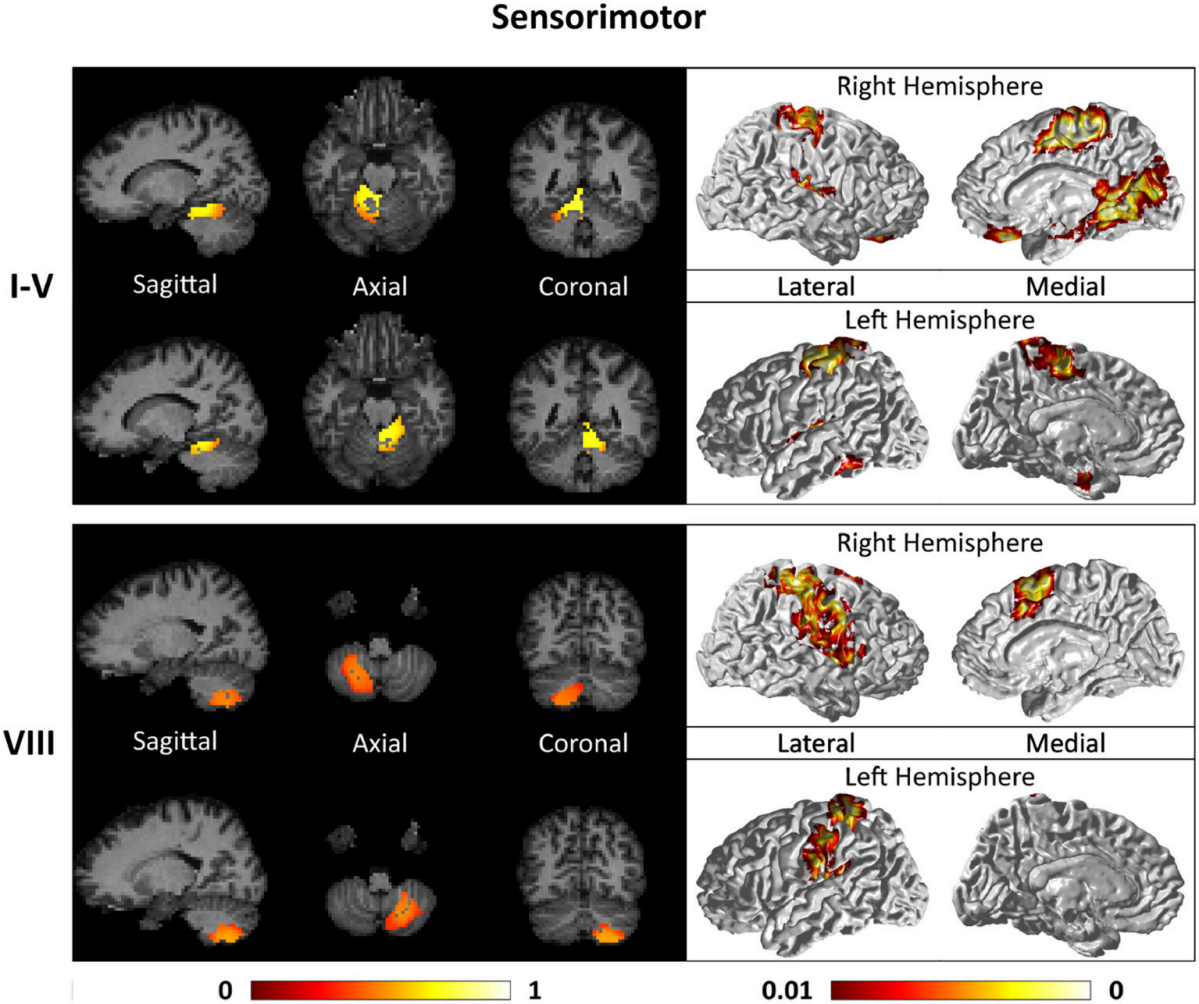
**Cerebellar clusters (left column) that correspond to lobules I–V (first row) and VIII (second row) are found to be functionally connected to sensory and motor cortical regions (right column)**.

**Figure 8:**
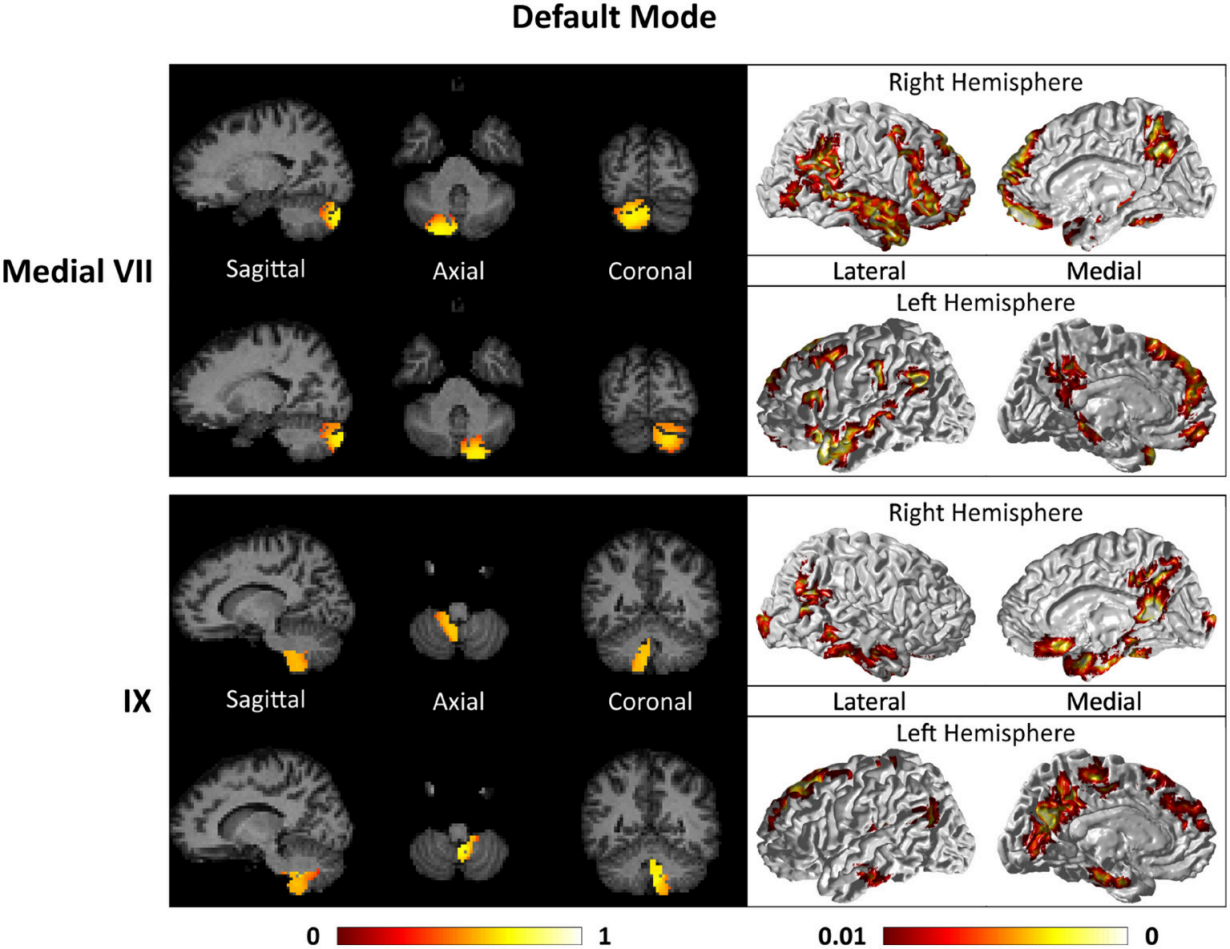
**Cerebellar clusters (left column) that correspond to lobules medial VII (first row) and IX (second row) are found to be functionally connected to cortical regions lying in the default mode network (right column)**.

**Figure 9:**
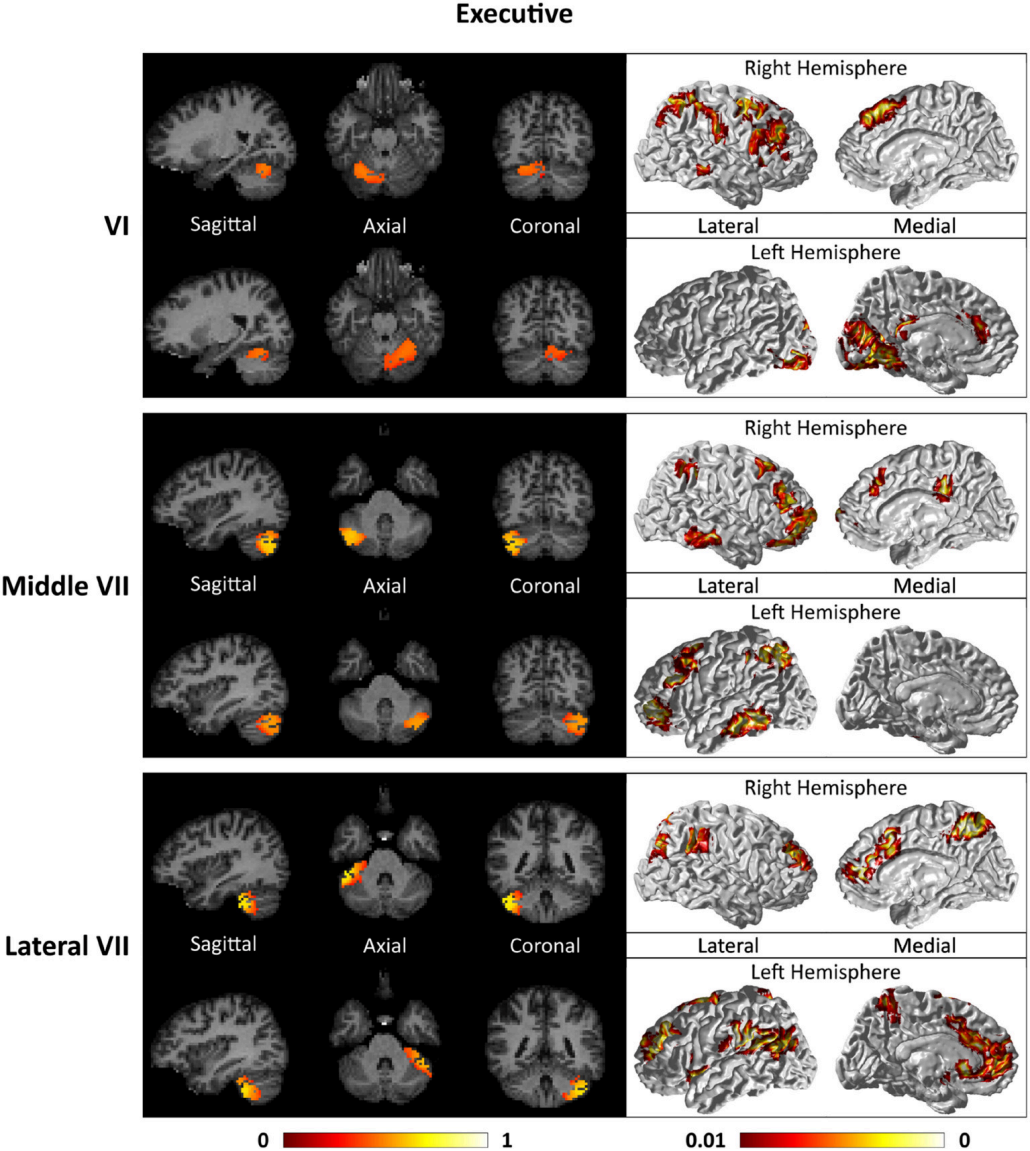
**Cerebellar clusters (left column) that correspond to lobules middle VII (first row) and lateral VII (second row) are found to be functionally connected to subnetworks of executive control and salience systems (right column)**.

Figures 7–**9** show the cerebello-cortical networks for all seven clusters. In these figures, the color in the cerebellar volume is an indication of the probabilities that the cerebellar voxels belong to this particular cluster as opposed to the other clusters, whereas the colors on the cortical surface is an indication of the *p*-values of the *t*-tests on β_*GLM*_. Only the cortical hemisphere that is on the opposite side to the cerebellar cluster is shown, since the cerebellar clusters showed predominantly contra-lateral connectivity to the cortex as expected biologically. Our cerebello-cortical networks mostly corroborate with findings of earlier studies (Habas et al., 2009; Krienen and Buckner, 2009; O'Reilly et al., 2010; Buckner et al., 2011; Bernard et al., 2012; Kipping et al., 2013).

For the motor cerebellum as represented by clusters in I–V and VIII, we found connectivity of the anterior (I–V) and inferior posterior (VIII) cerebellum with cortical regions lying in the sensorimotor system (Habas et al., 2009; Krienen and Buckner, 2009; Stoodley and Schmahmann, 2009; O'Reilly et al., 2010) as shown in Figure 7. Additionally we observe a stronger differentiation between motor (I–V) and somatosensory-related (particularly from left VIII) regions (Kipping et al., 2013). The connectivity of clusters in medial VII and IX was found with the prefrontal cortex (O'Reilly et al., 2010), as part of the default mode network (Habas et al., 2009; Krienen and Buckner, 2009; Buckner et al., 2011; Bernard et al., 2012) as depicted in Figure 8. Based on our cerebellar clustering we further observed that both clusters showed strong, but differentiated connectivity with the medial prefrontal cortex and precuneus, as well as the middle temporal cortex (Buckner et al., 2011). Furthermore, the connectivity of clusters in VI, middle VII and lateral VII was associated with subnetworks subserving functions of executive control and salience. In Figure 9, we illustrate that the medial-lateral clustering in VII is associated with a functional shift as indicated by their differential connectivity with fronto-parieto-(temporal) networks (Krienen and Buckner, 2009; Stoodley and Schmahmann, 2009; Buckner et al., 2011; Dobromyslin et al., 2012; Kipping et al., 2013).

A point to note is that the occipital cortex is implicated in both the cortical networks of left lobules I-V and right lobule VI. This is most likely introduced by the spatial smoothing of rs-fMRI signals of the cerebellum and the anatomically-adjacent occipital cortex (Buckner et al., 2011; Kipping et al., 2013).

## 4. Conclusion

In this paper, we introduced a new data-driven decomposition-based functional parcellation method, called Sparse Dictionary Learning Clustering (SDLC). SDLC outperforms other basic clustering techniques, including k-means clustering, hierarchical clustering with ward linkage, multi-class spectral clustering, temporal ICA and spatial ICA, based on the simulated data. SDLC was also used on real rs-fMRI data to parcellate the left and right cerebellum of 58 subjects each into seven clusters, as recommended by the stability analysis performed. When aggregated into a single group parcellation, the clusters obtained in the two hemispheres of the cerebellum were highly symmetrical. This suggests the validity of SDLC on the real data. The seven cerebellar clusters respectively comprised anatomical cerebellar lobules of I-V, VI, medial VII, middle VII, lateral VII, VIII, and IX. The functional connectivity between the cerebellar clusters and the cerebral cortex was similar to that in previous work (Habas et al., 2009; Krienen and Buckner, 2009; O'Reilly et al., 2010; Buckner et al., 2011; Bernard et al., 2012; Kipping et al., 2013). The clusters were identified as parts of the following systems: sensorimotor (lobules I–V and VIII), default mode (lobules medial VII and IX) and executive control (lobules VI, middle VII and lateral VII). Based on the differential connectivity patterns between the cerebellum and cortex, cerebellar clusters seem to contribute to a different degree to the above-mentioned brain systems. For example, clusters in medial VII and IX showed connectivity to cortical areas associated with the default mode system (Buckner et al., 2011). The functional implications of these non-motor cerebello-cortical systems remain to be investigated (Bellebaum and Daum, 2007).

There are other studies showing the cerebellar parcellations based on rs-fMRI. Buckner et al. (2011) obtained a cerebellar parcellation by utilizing information from the functional parcellation of the cerebral cortex. The cerebellar parcellation in Buckner et al. (2011) was obtained through cortical labels. The cerebellar voxels were assigned to the label of the cortical region that they have the strongest functional connection to. This is the direct opposite of what was done in this study, where the cerebellum was clustered directly and its functional connectivity to the cerebral cortex was determined. Although Buckner et al. (2011) also clustered the cerebellum into seven clusters, it is reasonable to expect that the results of Buckner et al. (2011) and this study would have some difference for this very reason, and indeed they seem to complement each other. Both approaches are important for the understanding of the functional connectivity of the cerebellum (Bernard et al., 2012). Another possible reason for explaining the difference between the cerebellar parcellation obtained in this study and those of other studies could be due to age range of individuals. While most studies use rs-fMRI data from adults, the rs-fMRI data for this study was acquired from children aged 6 years. The presence of wide-spread cerebello-cortical connectivity in children opens up the possibility of further analysis on the effects of development on the functional connectivity of the cerebellum.

We notice that the proposed SDLC did not incorporate spatial constraints. More sophisticated clustering techniques with spatial information, such as mean-shift, are definitely worth exploring in near future in the SDLC framework. However, it may be difficult to solve them via the multi-block hybrid proximal alternating method that provides a convergent solution. Even though our proposed SDLC does not impose any spatial constraint, we did not enter into problems that the cerebellar parcellation obtained was not spatially contiguous.

## Author contributions

AQ and CW contributed to the overall design of the study, method derivation and implementation, manuscript writing. JK contributed to the analysis of cerebello-cortical functional networks and their interpretation. CB and HJ contributed to the method derivation and fast algorithm.

## Funding

This research is supported by the Singapore National Research Foundation under its Translational and Clinical Research (TCR) Flagship Programme and administered by the Singapore Ministry of Healths National Medical Research Council (NMRC), Singapore- NMRC/TCR/004-NUS/2008; NMRC/TCR/012-NUHS/2014. Additional funding is provided by NMRC (NMRC/CBRG/0039/2013), and Singapore Ministry of Education Academic Research Fund Tier 2 (MOE2012-T2-2-130).

### Conflict of interest statement

The authors declare that the research was conducted in the absence of any commercial or financial relationships that could be construed as a potential conflict of interest.
